# Targeted therapy for hepatocellular carcinoma

**DOI:** 10.1038/s41392-020-00264-x

**Published:** 2020-08-11

**Authors:** Ao Huang, Xin-Rong Yang, Wen-Yuan Chung, Ashley R. Dennison, Jian Zhou

**Affiliations:** 1 Department of Liver Surgery and Transplantation, Liver Cancer Institute, Zhongshan Hospital, Fudan University, Shanghai, China; 2 Key Laboratory of Carcinogenesis and Cancer Invasion (Fudan University), Ministry of Education, Shanghai, China; 3 Shanghai Key Laboratory of Organ Transplantation, Zhongshan Hospital, Fudan University, Shanghai, China; 4 Department of Hepatobiliary and Pancreatic Surgery, University Hospitals of Leicester NHS Trust, Leicester, UK; 5 Institute of Biomedical Sciences, Fudan University, Shanghai, China; 6 State Key Laboratory of Genetic Engineering, Fudan University, Shanghai, China

**Keywords:** Gastrointestinal cancer, Cancer, Cancer therapy

## Abstract

The last 3 years have seen the emergence of promising targeted therapies for the treatment of hepatocellular carcinoma (HCC). Sorafenib has been the mainstay of treatment for a decade and newer modalities were ineffective and did not confer any increased therapeutic benefit until the introduction of lenvatinib which was approved based on its non-inferiority to sorafenib. The subsequent success of regorafenib in HCC patients who progress on sorafenib treatment heralded a new era of second-line treatment and was quickly followed by ramucirumab, cabozantinib, and the most influential, immune checkpoint inhibitors (ICIs). Over the same period combination therapies, including anti-angiogenesis agents with ICIs, dual ICIs and targeted agents in conjunction with surgery or other loco-regional therapies, have been extensively investigated and have shown promise and provided the basis for exciting clinical trials. Work continues to develop additional novel therapeutic agents which could potentially augment the presently available options and understand the underlying mechanisms responsible for drug resistance, with the goal of improving the survival of patients with HCC.

## Introduction

Primary liver cancer remains a major problem for all health care systems worldwide and is associated with a significant clinical, economic, and psychological burden. Hepatocellular carcinoma (HCC) accounts for ~90% of cases of non-metastatic tumors of the liver.^1^ During the past decades, research has shed light on the epidemiology, risk factors, and molecular and genetic profiles of HCC‚ contributing to the evolution of strategies for prevention, surveillance, early diagnosis, and treatment.^2,3^ Liver resection, ablation, and liver transplantation are potentially curative but require diagnosis at a sufficiently early stage. Unfortunately, a significant proportion of HCC patients present with intermediate and advanced stage disease often, despite diligent surveillance, and curative treatments are frequently not possible.^4^ In these patients, systemic therapy remains essential and its pivotal role and potential have stimulated considerable research over the past decade. In this review, we examine recent advances in targeted therapy and discuss the impact this has had on the management of HCC. We also provide an overview of the most important areas of HCC research including novel clinical trials and technical platforms which promise to facilitate substantial progress within the next decade.

## Approved first-line agents for HCC

### Sorafenib

**Fig. 1:**
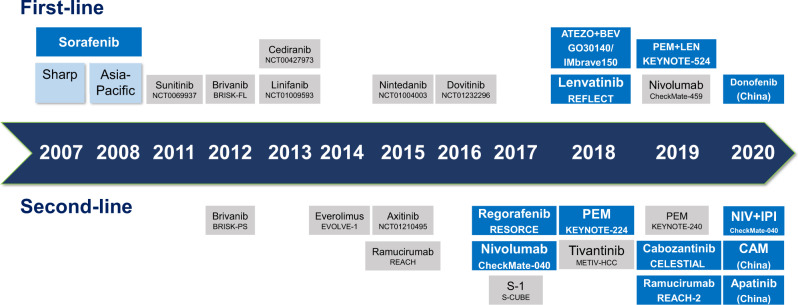
Overview of the targeted agents approved for HCC. ATEZO atezolizumab, BEV bevacizumab, CAM camrelizumab, LEN lenvatinib, PEM pembrolizumab, NIV nivolumab, IPI ipilimumab

The success of SHARP and Asia-Pacific trial promoted the approval of sorafenib as first-line targeted therapy for advanced HCC,^5–9^ ushering in the era of systemic treatment. Subsequently, virtually all trials were centered around sorafenib and it was used as a control with which novel first-line agents were compared and evaluated in an attempt to improve the prognosis of patients with HCC. Unfortunately, despite a number of trials which compared these novel agents including sunitinib,^10^ brivanib,^11^ cediranib,^12^ linifanib,^13^ dovotinib,^14^ and immune-checkpoint inhibitors (ICIs) to sorafenib, none achieved the predefined primary end points (Fig. 1). In addition, during the decade when these agents were investigated, the median overall survival (OS) of sorafenib monotherapy as first-line treatment for advanced HCC increased from 10.7 months (SHARP) to 14.7 months (CheckMate-459), further consolidating its position. Meanwhile, the anti-tumor activity and safety of sorafenib have been validated in real-world setting. Subanalyses of the SHARP and Asia-Pacific trials found sorafenib was effective and safe irrespective of disease etiology, disease burden, ECOG (Eastern Cooperative Oncology Group performance status) status, etc.^15–17^ The safety of sorafenib was consistent across Child-Pugh A and B patients in clinical practice^18^ and the occurrence of side effects such as hand-foot syndrome and diarrhea were associated with an improved OS.^19^ Baseline hepatic function, clinicopathological factors, and etiology also affect the prognosis in HCC patients treated with sorafenib.^20^ In addition, sorafenib exerts anti-tumor effects with recurrent tumors following liver transplantation, conferring a survival advantage when compared with best supportive care (BSC).^21–23^ Noticeably, the application of sorafenib in clinical practice displays significant regional variations and incompliance with guidelines besides its usage as first-line therapy. It is common that initially unresectable HCCs got downstaged after sorafenib treatment and underwent curative-intent surgery^24–28^ and locoregional therapies before sorafenib were commonly encountered in real-world settings.^29,30^

The clinical benefit of sorafenib however remains modest and the complex molecular pathogenesis of HCC stimulated the investigation of combinations of sorafenib with other molecular targeting drugs. Sorafenib has been combined with anti-angiogenic agents, MEK/ERK pathway inhibitors, mTOR pathway inhibitors, histone deacetylase inhibitors, EGF/EGFR pathway inhibitors, and HGF/c-Met pathway inhibitors.^31^ Other agents such as interferon,^32^ selumetinib,^33^ capecitabine,^34^ tegafur-uracil,^35^ gemcitabine and oxaliplatin (GEMOX),^36,37^ and gemcitabine alone^38^ have also been evaluated but to date no treatments involving combinations containing sorafenib have succeeded in phase III trials.

Since sorafenib and TACE are both recommended therapies for advanced HCC, it is reasonable to expect that their combined use would confer benefits when compared with monotherapy. Results however highlighted regional differences and the heterogeneity of the trial protocols. In TACE 2, the multi-center, randomized, placebo-controlled, phase 3 European trial, when compared with TACE alone the addition of concurrent sorafenib, unlike the SPACE trial,^39^ did not improve progression free survival (PFS).^40^ It was also shown that the addition of sorafenib did not confer any survival benefit in patients with unresectable HCCs, who had already responded to TACE.^41^ Contrasting with these findings, retrospective studies from China have shown that combination therapy with sorafenib and TACE increased OS by more than 50% compared with TACE alone,^42–53^ which was supported by the findings from a number of other groups.^54,55^ Recently, the TACTICS trial, a randomized, multi-center prospective trial from Japan reported an improved PFS for TACE plus sorafenib compared with TACE alone (25.2 vs 13.5 months; *p* = 0.006),^56^ although this trial used a redefined PFS (not conventional PFS but time until “unTACEable” progression). The TACTICS trial also used time to any cause of death plus OS as primary endpoints (results not reported) and compared with sorafenib monotherapy, TACE plus sorafenib was only superior in controlling tumor progression and did not prolong OS.^57,58^

The acceptance of sorafenib as the standard to which other newer agents and non-surgical interventions are compared has resulted in studies comparing its use as monotherapy with TACE plus external beam radiotherapy^59^ and TACE plus intensity-modulated radiotherapy combined with sorafenib.^60^ In the SARAH study, selective internal radiotherapy with yttrium-90 resin microspheres did not produce any survival benefit compared with sorafenib in locally advanced and inoperable HCC (median OS, 8.0 vs 9.9, *p* = 0.18), and did not meet the primary endpoint of OS.^61^ Similarly, the addition of selective internal radiation therapy to sorafenib did not result in a significant improvement in OS compared with sorafenib alone.^62^ Bettinger et al.^63^ however did demonstrate that stereotactic body (external beam) radiotherapy employed as monotherapy (SBRT) was able to improve OS compared with sorafenib and SBRT with TACE also provided improved OS and PFS when compared with sorafenib and TACE in combination.^64^ In a recent trial of hepatic arterial infusion chemotherapy (HAIC; NCT02774187), He et al.^65^ reported that sorafenib plus HAIC with FOLFOX improved OS compared with sorafenib alone in advanced HCC when portal vein invasion was present, which was supported by other studies.^66,67^ Although the SCOOP-2 trial found sequential HAIC with cisplatin and sorafenib did not improve the survival benefit compared with sorafenib alone this is likely to have resulted from the study being underpowered for the primary and secondary endpoints.^68^

Due to the high recurrence rates following hepatectomy for HCC, approaches to adjuvant therapy has been extensively investigated although previous attempts, including the use of anti-viral agents, have been largely unsuccessful. Based on the palliative use and success of sorafenib its potential in the adjuvant setting was investigated and improved survivals following surgery anticipated. Unfortunately, this has not been demonstrated and it failed to reduce postoperative tumor recurrence in the STORM trial^69^ and other western studies.^70^ Explanations for the negative outcome in the STORM trial include high-dose modification rates, short treatment durations, and the enrollment of patients who were not at high risk of tumor recurrence (91% with no evidence of tumor satellites, 91% with one lesion, and 68% with no microscopic vascular invasion).^71^ Consistent with this viewpoint, Wang et al.^72^ reported no case of recurrence during the sorafenib dosing period whereas 4/14 patients suffered recurrence of their tumor within 7 months of discontinuation of sorafenib^72^ and persistent sorafenib intake following postoperative recurrence improved OS.^73^ Considering that patients who respond to sorafenib may belong to limited clinical or biological subsets, the effectiveness of sorafenib in an unselected population cohort supports its use in the adjuvant setting. A number of studies from the Far East including China, Japan, and Korea include patients with HCCs who are treated with hepatectomy despite their tumors being outside Barcelona Clinic Liver Cancer Classification (BCLC) guidelines, and although the results are difficult to compare due to heterogeneity of the protocols the results are positive. Sorafenib significantly reduces tumor recurrence in BCLC stage C patients^74,75^ and increases disease-free survival (DFS),^76^ and Zhuang et al.^77^ demonstrated that adjuvant therapy increased disease free survival (DFS) and OS. Sorafenib treatment following hepatectomy significantly prolonged the OS of advanced HCC rather than intermediate HCC.^78^ In addition to BCLC stage, patients who underwent hepatectomy and were pathologically diagnosed with microvascular invasion (MVI) also benefited from adjuvant sorafenib treatment.^79^ In line with these results, a large recent study with propensity score matching analysis also demonstrated that sorafenib significantly improved overall and recurrence-free survival following resection.^80^ The results from these studies which include all eligible patients suggest that more precise stratification would enable the identification of those patients who will benefit most from the use of adjuvant sorafenib and those in where additional treatment is not appropriate. Ongoing trials are attempting to evaluate the role of sorafenib in patients with MVI following radical resection (NCT02867280 and NCT02537158).

### Lenvatinib

Following the approval of sorafenib for use in the treatment of HCC it takes more than a decade before the second first-line targeted agent for HCC emerged. Lenvatinib was approved for the first-line therapy in advanced HCC following the results of the REFLECT trial, a randomized phase III non-inferiority trial published by Kudo et al.^81^ Although not approved for long, further multi-center data from “real-world conditions” confirmed the efficacy of lenvatinib, regardless of previous tyrosine kinase inhibitor (TKI) therapies^82,83^ and lenvatinib monotherapy demonstrated anti-tumor activity for more than 4 years in unresectable HCC when portal vein invasion was present.^84^ In intermediate-stage HCC patients with tumors exceeding the up-to-seven criteria, for whom TACE is not helpful, lenvatinib could provide significant longer OS (37.9 vs. 21.3 months) and PFS (16.0 vs. 3.0 months).^85^ Lenvatinib pharmacokinetics in HCC is affected by body weight^86,87^ and a sufficient dose (relative dose intensity, RDI) is required to achieve a good therapeutic effect and consequently improved outcomes and prognosis are associated with the preservation of liver function which reduces the number of patients who need to discontinue their treatment.^88–91^ With lenvatinib, unlike other TKIs, there are issues with thyroid toxicity and surveillance for thyroid abnormalities during treatment is important.^92^ Hypothyroidism is not unusual and there are also fewer common reports of thyrotoxicosis and destructive thyroiditis.^93^ From a health economics standpoint however, lenvatinib is more cost effective than sorafenib.^94,95^

### Second-line targeted agents for HCC

Still, sorafenib displays limited anti-tumor activity and some initially sorafenib-sensitive would eventually succumb to the disease, indicating the acquired resistance to sorafenib reduces its beneficial effects and an urgent need for second-line therapy.

### Regorafenib

Initial attempts to discover effective second-line agents were unsuccessful and mirrored attempts to develop first-line agents which were superior to sorafenib.^96^ The RESORCE trial was a randomized, double blind, placebo-controlled, and phase III trial demonstrating the effectiveness of regorafenib in patients who had progressed on sorafenib treatment. This study finally confirmed the potential of second-line agents and ushered in the era of second-line and sequential therapy.^97^ Regorafenib provided survival benefit regardless of the rate of disease progression during prior sorafenib treatment or since the last sorafenib dose.^98^ This was confirmed by Yoo et al.^99^ in a retrospective study of safety and efficacy in Korean patients where data were consistent with those from the RESORCE trial. Regorafenib was even shown to be effective in patients with HCC recurrence following liver transplantation with a median OS of 12.9 months following regorafenib initiation and 38.4 months following sorafenib initiation (95% CI, 18.5–58.4) for the sorafenib followed by regorafenib sequential therapy.^100^

However, not all patients who progress on sorafenib are candidates for second-line therapy.^101^ In clinical practice only ~30% of patients are eligible for second-line regorafenib treatment.^102^ Good liver functional reserve and ECOG performance status during sorafenib treatment contributed to the efficacy and better outcomes of subsequent treatment,^103,104^ including lenvatinib.^105^ This may in part be due to the RDI required to achieve a clinically significant improvement in prognosis.^106^ This is supported by the demonstration that the new liver reserve function biomarker, albumin-bilirubin grade (ALBI),^107^ successfully identified regorafenib candidates and that in the selected cohort a median OS of 15.6 months was achieved compared with 6.8 months for non-candidates.^108^ Even in patients not eligible for regorafenib, the ones with an ECOG-PS score of 0, the absence of MVI, and TTP (time to progression) ≥4 months could still have acceptable postprogression survival.^109^ Long-term treatment with regorafenib has also been shown to reduce angiogenesis and improve portal hypertension (PHT) and acute administration ameliorates portal haemodynamics, suggesting that it may be especially suitable for patients with PHT and preserved liver function.^110^

### Cabozantinib

Cabozantinib is another small molecule inhibitor of the tyrosine kinases which are implicated in the progression of HCC and the acquired resistance to sorafenib. Cabozantinib blocks the receptors involved in oncogenesis and angiogenesis including VEGFR 1, 2, 3, hepatocyte growth factor receptor (MET), AXL and the angiopoietin receptors TIE-2, RET, c-Kit and FLT-3 in vitro and in vivo. In CELESTIAL trial, cabozantinib achieved the primary endpoint with median OS of 10.2 months compared with 8.0 months for the placebo group^111^ and was consequently approved in the EU and USA. There remains a paucity of data however from real-world clinical practice examining the sequential treatment utilizing cabozantinib as the second-line agent, it is a costly option associated with frequent high-grade adverse events. Consequently, several studies have addressed the cost-effectiveness of cabozantinib using the cost and utility data extracted from the CELESTIAL trial. The conclusion from these studies is consistent and confirms that at its current cost point, the gain of quality-adjusted life-years for cabozantinib (QALYs, 0.067–0.16) and the incremental cost-effectiveness ratio (ICER, $156 437–$1,040,675) mean that it is not a cost-effective treatment option for patients with sorafenib-refractory HCC,^112–114^ compared with regorafenib (QALY, 0.18–0.25 and ICER, $201,797–$224,362).^115,116^

### Ramucirumab

Ramucirumab is a fully human recombinant IgG1 monoclonal antibody targeting the VEGF2 receptor. Although ramucirumab failed to meet its primary endpoint as second-line treatment in the REACH trial,^117^ subgroup analysis found survival benefit in patients with AFP of 400 ng/ml or higher.^118–121^ This was later confirmed in the REACH-2 trial,^122^ which led to the approval of ramucirumab as second-line treatment for advanced HCC. REACH-2 is the first positive phase 3 trial in patients with HCC performed in a biomarker-selected patient cohort and more recent findings demonstrated that AFP-enriched HCCs displayed significant activation of VEGF which suggests the underlying mechanism of action and confirms the potential value of biomarker-driven clinical trials.^123^

### Immune checkpoint therapy and TKI inhibitors

ICIs stand as the mainstream of immunotherapy. The CheckMate-040^124^ and KEYNOTE-224^125^ studies evaluated the safety and efficacy of nivolumab and pembrolizumab in patients with advanced HCC refractory to previous sorafenib treatment, which established the basis for accelerated approval by the FDA as second-line treatment. Subanalysis of CheckMate-040 data validated the safety and efficacy of nivolumab in Asian cohort.^126^ In an international real-world cohort study, ICIs have showed promising efficacy and safety in advanced HCCs as systemic first-/second-/third-/fourth-line treatment, with median OS and PFS of 11.0 and 4.6 months respectively^127^ and an excellent response to anti-PD-1 therapy has also been described in case report.^128^ Although the subsequent phase III KEYNOTE-240 trial did not meet its pre-specified statistical significance in respect of improved PFS and OS, the results were consistent with previous KEYNOTE-224.^129^ The KEYNOTE-394 presently underway in Asian patients may clarify the role of pembrolizumab in cases of advanced HCC with a viral background (NCT03062358). Recently, CheckMate-459, the multi-center phase III randomized sorafenib controlled trial evaluating nivolumab as first-line treatment for advanced HCC, failed to achieve its endpoints (ESMO 2019) but nivolumab did prolong OS (16.4 vs. 14.7 months) and achieve long-time disease control, less adverse events (AEs) and survival benefit regardless of the level of PD-L1 expression. Furthermore, nivolumab improved the survival of HCC patients whose etiology was HBV/HCV and did not reactivate hepatitis. Camrelizumab (SHR-1210, Hengrui Pharmaceutical), is an anti-PD-1 inhibitor from China investigated for the treatment of Hodgkin lymphoma and HCC. It has been shown to have antitumor activity in previously treated Chinese patients with advanced HCC in a multi-center, open-label, parallel-group, randomized, phase II trial (NCT02989922),^130^ providing evidence for the effectiveness of PD-1 therapy for HBV related HCC in Chinese patients. The results from other trials investigating novel ICIs including durvalumab, avelumab, tislelizumab, sintilimab, tremelimumab, ipilimumab, spartalizumab, and toripalimab will hopefully yield positive results and provide further options for the treatment of patients with HCC, particularly those who have relapsed on first-line treatments.

Further efforts to enhance the treatment effect of ICIs include dual ICIs treatment and combination therapy of ICIs with other kinds of targeted agents. For dual ICIs treatment, the initial results from CheckMate 9DW were astonishing: the objective response rate was 32%, higher than monotherapy of any ICIs alone. FDA has approved nivolumab in combination with ipilimumab for patients with HCC previously treated with sorafenib. Treatment modalities such as radiotherapy and anti-angiogenesis agents which affect antigen release or modulate the tumor microenvironments have the potential to increase the efficacy of immunotherapy and the combination of targeted agents with ICIs are attracting the attention of a number of research groups and in vitro studies and early-phase clinical trials assessing combination treatments have shown promising anti-tumor effects in patients with advanced HCC. In vitro evidence by Qui et al.^131^ demonstrated that lenvatinib and regorafenib could affect the expression of PD-L1 and real-time PCR results suggested that the mRNA expression of PD-L1 in the lenvatinib group was significantly higher than that in the control group, while its expression in the regorafenib group was significantly lower. When combined with anti-PD-1, lenvatinib can modulate cancer immunity in the tumor microenvironment and enhance antitumor activity.^132,133^ In July 2019, the FDA announced its approval of the first combination therapy employing the TKI lenvatinib with the ICI pembrolizumab based on the results from the KEYNOTE-524/Study 116 (NCT03006926) for the treatment of HCC. Recently, results from Study 117 (Phase Ib, NCT03418922) showed marginally better results for lenvatinib with nivolumab than lenvatinib with pembrolizumab. MET-mediated phosphorylation leads to a decreased expression of PD-L1 using the combination of MET inhibitors tivantinib and capmatinib, anti-PD1 and anti-PDL1 produced an additive effect which slows the growth of HCCs in mice.^134^ Clinically, based on the results from the experimental arm A of the GO 30140 study (NCT02715531), the FDA approved atezolizumab plus bevacizumab as breakthrough therapy for untreated advanced or metastatic HCC.^135^ Individual case studies also reported promising results for the use of combined TKI and anti-PD1/PD-L1 agents for advanced HCC.^136–138^ Such results were confirmed in the phase III trial IMbrave 150 study (NCT03434379) which reported that atezolizumab combined with bevacizumab resulted in better OS and PFS than sorafenib in patients with unresectable HCC.^139^ Other combination therapies include Galunisertib with nivolumab (NCT02423343), spartalizumab with and without capmatinib (NCT02795429), FGF401 with spartalizumab (NCT02325739), regorafenib with pembrolizumab (NCT03347292), cabozantinib with nivolumab (NCT03299946), avelumab with axitinib (NCT03289533), ramucirumab with durvalumab (NCT02572687), and XL888 with pembrolizumab (NCT03095781; Table 1).

**Table 1:** Trials investigating the combination therapy of ICIs and TKIs in HCC

Trial name/identifier	Patient No.	Study type	Line	Interventions	Primary endpoint	Study status
LEAP-002/NCT03713593	1097	Phase III	First	Lenvatinib + pembrolizumab vs lenvatinib	PFS+OS	Active, not recruiting
CheckMate 9DW/NCT04039607	1084	Phase III	First	Nivolumab + ipilimumab vs lenvatinib or sorafenib	OS	Ongoing
COSMIC-312/NCT03755791	740	Phase III	First	Cabozantinib + atezolizumab vs sorafenib	PFS+OS	Ongoing
ORIENT-32/NCT03794440	566	Phase III	First	Sintilimab + IBI305 vs sorafenib	OS, ORR	Ongoing
SHR-1210-III-310/NCT03764293	448	Phase III	First	Camrelizumab + apatinib vs sorafenib	OS+PFS	Ongoing
SHR-1210-III-305/ NCT03605706	448	Phase III	First	Camrelizumab + apatinib vs FOLFOX 4 or sorafenib	OS	Ongoing
IMMUNIB/NCT03841201	50	Phase II	First	Nivolumab + lenvatinib	ORR, AE	Ongoing
NCT03439891	40	Phase II	First	Nivolumab + sorafenib	MTD, ORR	Ongoing
NCT03211416	27	Phase Ib/ II	First	Sorafenib + pembrolizumab	ORR	Ongoing
KEEP-G 04/NCT04052152	20	Phase II	First	Anlotinib + sintilimab	ORR AE	Ongoing
VEGF Liver 100/NCT03289533	22	Phase Ib	First	Avelumab + axitinib	AE	Completed
KN/743/NCT03347292	57	Phase I	First	Regorafenib + pembrolizumab	AE	Ongoing
GOING/NCT04170556	60	Phase II	Second	Regorafenib + nivolumab	AE	Ongoing
REGOMUNE/NCT03475953	/^a^	Phase I/II	Second	Regorafenib + avelumab	CR, PR	Ongoing
NCT02423343	/^a^	Phase Ib/ II	Second	Galunisertib + nivolumab	Phase Ib: MTD	Ongoing

*PFS* progression-free survival, *OS* overall survival, *ORR* objective response rate, *AE* adverse events, *MTD* maximum tolerated dose, *CR* complete response, *PR* partial response

^a^Trials enroll not only HCC patients

Immune-related adverse events (IRAEs) occur frequently during treatment with ICIs and the clinical consequences can be significant.^140^ Activation of the immune system leads to damage of normal healthy tissues and IRAEs can have myriad effects and involve a number of different organs and have been reported to produce colitis, hepatitis, pneumonitis, dermatitis, myocarditis, endocrine glands inflammation, and rheumatic and musculoskeletal phenotypes including inflammatory arthritis, arthralgia, myositis, and sicca syndrome.^141^ Although the precise pathophysiology underlying the IRAEs side effects during treatment with ICIs remains unknown, discontinuing administration and the use of steroids is generally effective. In severe cases, however, additional immunosuppressants may be required but based on current available evidence, immunosuppression for IRAEs does not appear to compromise the antitumor response to the ICI treatment.^142,143^

### Promising agents and treatment regimens

Despite abovementioned targeted drugs, novel agents have been continuously under development (Table 2). Of note, apatinib, a novel inhibitor of VEGFR2 tyrosine kinase, has attracted considerable attention and there is now a significant body of work describing clinical experience with its use. Although less effective than sorafenib as a first-line treatment in a retrospective study,^144^ apatinib still displayed promising anti-tumor effects in sorafenib-resistant HCC,^145–147^ where portal vein invasion was present,^148^ when metastases have occured,^149,150^ and for unresectable and relapsed HCCs.^151,152^ Combination therapy in studies utilising apatinib with TACE have achieved better clinical effectiveness than TACE alone, with tolerable AEs.^153–161^ Recently, the combination of apatinib with the anti-PD-1 monoclonal antibody camrelizumab achieved partial response rates of 50%.^153^ The results of other ongoing trials including the phase III trial comparing TACE and apatinib with sorafenib as first-line treatment for locally advanced or metastatic and unresectable HCC (NCT 03764293) and the adjuvant apatinib after hepatectomy for the prevention of tumor recurrence (NCT03722875 and NCT03261791) will hopefully prove effective and add to the presently available therapeutic options.

**Table 2:** Trials investigating targeted therapy in advanced HCC

Trial name/identifier	Patient no.	Study type	Line	Interventions	Primary endpoint	Study status
IMbrave150/ NCT03434379	480	Phase III	First	Atezolizumab + bevacizumab vs Sorafenib	OS, PFS	Completed
ZGDH3/NCT02645981	668	Phase II/III	First	Donafenib vs sorafenib	OS	Completed
HIMALYYA/ NCT03298451	1310	Phase III	First	Durvalumab + tremelimumab vs sorafenib	OS	Ongoing
RATIONALE-301 / NCT03412773	674	Phase III	First	Tislelizumab vs sorafenib	OS	Ongoing
PHOCUS	600	Phase III	First	Pexa-Vec + sorafenib vs sorafenib	OS	Ongoing
NCT04344158	648	Phase III	First	AK105 + anlotinib vs sorafenib	OS	Ongoing
ALTER0802/ NCT02809534	60	Phase II	First	Anlotinib	PFS 12W	Ongoing
AHELP/NCT02329860	400	Phase III	Second	Apatinib vs placebo	OS	Completed
KEYNOTE-394 / NCT03062358	450	Phase III	Second	Pembrolizumab + BSC vs placebo + BSC	OS	Ongoing
NCT04080154	28	Phase II	Second	Anlotinib	PFS	Ongoing

*OS* overall survival, *PFS* progression-free survival, *BSC* best supportive care

These promising results have stimulated the investigation of other new agents, the combinations of agents and regimens, which have been thoroughly discussed in a recent review from Zhu and Sun.^154^ The combination of bevacizumab and erlotinib has been extensively evaluated as first-^155^ or second-line in advanced HCCs,^156–162^ but unfortunately the heterogeneous nature of the results precludes firm conclusions and recommendations. Recently, a single-arm meta-analysis of prospective studies found that combination therapy with bevacizumab and erlotinib used as second-line treatment was associated with a favorable PFS (16 weeks, *P* = 0.012) and OS (12 months, *P* = 0.048), suggesting that future well-designed and sufficiently powered large-scale RCTs should be able to identify the potential contribution of these agents.^163^

Preclinical evidence for cyclin-dependent kinase (CDK) targeting therapies in HCC has showed promise and supports their investigation,^164–166^ especially with the potential ability to abrogate the emergence of sorafenib resistance^167^ and sensitize HCC to regorafenib treatment.^168^ A number of CKD inhibitors are presently undergoing evaluation including palbociclib (NCT01356628), milciclib (NCT03109886), and ribociclib (NCT02524119). The anti-MET monoclonal antibody emibetuzumab exhibited the greatest antitumor activity in HCC when combined with ramucirumab and had an excellent safety profile^169^ and for HCC with high MET expression there was an almost 3-fold increase in PFS (8.1 vs. 2.8 months) relative to those with low MET expression, suggesting the potential for further biomarker-driven clinical trials. Rigosertib is a synthetic benzyl styryl sulfone small molecule inhibitor which has been used in the treatment of monomyelocytic leukemia and due to its activity as a RAS- and PLK1-signaling inhibitor, it was investigated in HCC patients who demonstrate upregulation of PLK1 during tumor development and HRAS expression in advanced HCC. High expression levels of PLK1 are also significantly correlated with poor patient survival and the multiple effects of rigosertib could be beneficially employed to produce a therapeutic “dual-hit” approach in selected patients.^170^ Donafenib is a novel multi-kinase inhibitor which is similar to sorafenib, displaying comparable or better safety and efficacy when treating advanced HCC in phase 1b trial and phase 3 studies using sorafenib as the control (NCT02645981).^171^ There are ongoing trials evaluating novel agents such as anlotinib, another multi-kinase inhibitor which is orally administered and targets VEGFR, fibroblast growth factor receptor (FGFR), platelet-derived growth factor receptors (PDGFR), and c-kit (NCT02809534). Tivozanib is another oral inhibitor of VEGFR-1/2/3 with promising activity against HCC in vivo (NCT01835223) and TRC105, which despite demonstrating clinical activity and being well tolerated in HCC patients following sorafenib, has not to date met prespecified criteria and its development in HCC continues as combination therapy with sorafenib (NCT02560779).

### Biomarker-driven targeted therapy

Despite extensive research investigating potential biomarkers to aid the development of protocols for the treatment of HCC, none have so far been identified to be able to predict the effect of, or response to treatment with sorafenib.^172–182^ Although the molecular classification of HCC has been widely reported (Table 3) to date it remains unclear whether this basic genomic and proteomic data will prove valuable in guiding targeted therapies.^183–190^

**Table 3:** Molecular classification of HCC

Researcher	Year	Classification	Type	Case no.
Boyault et al.	2007	G1–G6	Transcriptome	57
Hoshida et al.	2009	S1–S3	Transcriptome	603
Schulze et al.	2015	Msig 1–6	Exome sequencing	243
/	2017	iC1–iC3	Multiomocis	363 + 196
Sia et al.	2017	25% HCCs with adaptive or exhausted immune responses	Immune cell profiling	956
Kurebayashi et al.	2018	Immune-high, -mid, and –low	Immuno-microenvironment	158
Shinata et al.	2019	MS-1, −2-, −3	Transcriptome and gonome	183
Jiang et al.	2019	S-I, S-II and S-III	Proteomics	110

The continued belief that the future lies with personalized treatment which will be made possible through the rapid developments in next generation sequencing and the precision medicine that it underpins, have encouraged the development of novel trial designs.^191^ These novel trials designs offer new hope that biomarker-driven targeted therapies can be modulated and tailored on an individual basis.^192,193^ Using the prospectively archived tumor tissue and baseline plasma samples from HCC patients receiving regorafenib in the RESORCE trial, it was found the plasma miRNA panel and genetic mutational signatures in tumors were able to predict the response to regorafenib.^194^ In BIOSTORM, the biomarker companion study of STORM, multigene signatures associated with improved RFS with adjuvant sorafenib treatment after hepatectomy were identified and in the future could be used to guide treatment protocols.^195^ This approach is supported by case series where patients with *CDKN2A*-inactivating, *CTNNB1*-activating, *PTEN*-inactivating, and *MET*-activating mutations received palbociclib (CDK4/6 inhibitor), celecoxib (COX-2/Wnt inhibitor), sirolimus (mTOR inhibitor), and cabozantinib, respectively, with a reduction of des-gamma-carboxy prothrombin and AFP following treatment.^196^

Approaches to developing biomarker-driven targeted therapy strategies have also been examined in vitro and inactivating mutations in *TSC1* and *TSC2* have been shown to confer sensitivity to the mTOR protein. Aurora kinases are known to be oncogenic and overexpressed in a variety of tumors including colon, breast and prostate cancer and HCC tumor and other genetic mutations are known to affect the response to tyrosine kinase inhibitors and amplification of the *MET* gene is associated with hypersensitivity to cabozantinib which inhibits the tyrosine kinases c-Met and VEGFR2.^197,198^ The Wnt/β-catenin and Akt/mTOR pathways have been investigated and are reported to be co-activated in 14.4% of HCCs with the result that inhibition of the Jak/Stat pathway has therapeutic potential.^199^ Considering the wide range and type of genetic alterations which may act as potential targets,^185^ it is reasonable to believe that with an appropriate and well-designed trial protocol it should be possible to identify and validate specific biomarkers which will predict the response to specific targeted agents. Thus, we can prevent the use of treatments which can have no therapeutic effect, and may be associated with significant, avoidable toxicity.

### Drug resistance of targeted therapy for HCC

**Fig. 2:**
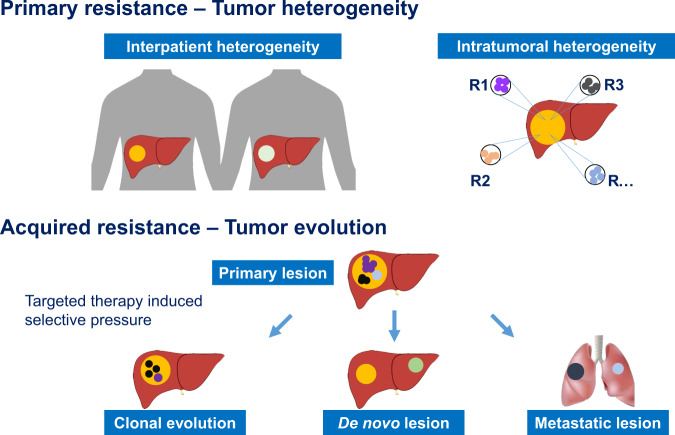
The primary drug resistance mainly derives from interpatient and intratumoral heterogeneity while tumor evolution during treatment leads to spatial- and temporal-heterogeneity, which cause acquired resistance. Tumor heterogeneity and clonal evolution stands as the main reasons for targeted drug resistance

Drug resistance remains the principle cause of treatment failure during the use of targeted therapies^200^ and tumor heterogeneity and clonal evolution are the underlying mechanisms (Fig. 2), with the former mainly involved in primary resistance and the later acquired resistance.^201^ HCC is a remarkably heterogeneous disease exhibiting inter-patient heterogeneity, intertumoral heterogeneity among multifocal tumors and intratumoral heterogeneity (ITH) within tumors. This heterogeneity explains the attraction of targeted therapies and the search for biomarkers which can reliably predict the response to different agents. Nevertheless, it is also clear that the potential for this degree of heterogeneity renders the task of identifying single useful biomarkers or combinations of biomarkers extremely complex. On the other hand, the heterogeneity that has been identified does explain the, often quite dramatic, differences in the response to different therapeutic agents or combinations of agents.

Unlike other primary cancers, multifocal lesions in liver is not uncommon whether derived from synchronous carcinogenesis or intrahepatic metastases. When multiple tumors occur, despite the fact that they originate from genetically similar cells and the potential etiology is the same, the lesions differ significantly (often very significantly) from each other demonstrating genomic alterations, varied biological behavior and loco-regional tumor microenvironment diversity which produces the well described differences in response to therapeutic agents.^202,203^ Within the tumor, intratumoral heterogeneity (ITH) has been found at genomic,^204^ epigenetic,^205^ transcriptional^206^, and protein level.^207,208^ Considering the level and extent of the intratumoral heterogeneity it is not surprising that the response to single treatment is equally heterogenous and irrespective of the efficacy of a single agent the most favorable outcome that can be reasonably expected is the eradication of a small portion of the total tumor cell burden leaving resistant clones surviving and responsible for progression.^209^ Although trunk mutational events in HCC are less heterogeneous^210^ and it is reported that single region sample could effectively recapitulate the genomic or proteomic features of HCC,^211–213^ which seems to shed light on overcoming drug resistance by ITH, tumor evolution due to selective pressure from targeted therapy brings new challenges.^214^ Thus, we have to find new ways to circumvent tumor heterogeneity and tumor evolution.

Fortunately, several novel techniques including single-cell sequencing, liquid biopsy, circulating tumor DNA (ctDNA), patient-derived cell-lines (PDC), patient-derived organoid (PDO), and patient-derived xenografts (PDX) should enable us to track cancer evolution in HCC. Compared with bulk tumor tissue sequencing, single-cell sequencing provides higher sensitivity and specificity, delineating tumor biology and characterization of cancer stem cell heterogeneity to understand the cellular diversity of HCC.^215^ Single-cell whole-genome sequencing has been used to examine the diverse modes of clonal evolution in HCC and relate this to tumor morphology. Variations are seen to occur early in tumor development but subsequently demonstrate stability during tumor progression. These findings suggest that treatment strategies could be developed based on the knowledge of tumor morphology.^216^

ctDNA represents a non-invasive, dynamic method to profile the tumor genome, predict treatment response, monitor disease progression, and help elucidate the mechanisms of drug resistance.^217^ In gastrointestinal cancers, ctDNA has been shown to outperform single-lesion tumor biopsies in discovering alterations which produce clinically relevant resistance and the mechanisms responsible for resistance to multiple agents.^218^ Novel alterations and parallel evolution which result in treatment-associated resistance are common in ctDNA, although the occurrence of multiple treatment related alterations in the circulation confound attempts to integrate the results into clinical protocols.^219^ When tumor biopsies are not available genetic profiling is possible using ctDNA which can provide a similar level of accuracy in respect of identifying somatic mutations and has the added advantage of being able to also detect de novo mutations.^220^ To date, few studies had examined the value of ctDNA to guide targeted therapy in HCC patients although preliminary results have demonstrated the feasibility of ctDNA in circumventing ITH,^212^ delineating tumor evolution,^221,222^ efficiently capturing mutations indicative of targeted therapy,^196,223^ and dynamically revealing genomic change during pharmacological treatment.^224^

**Fig. 3:**
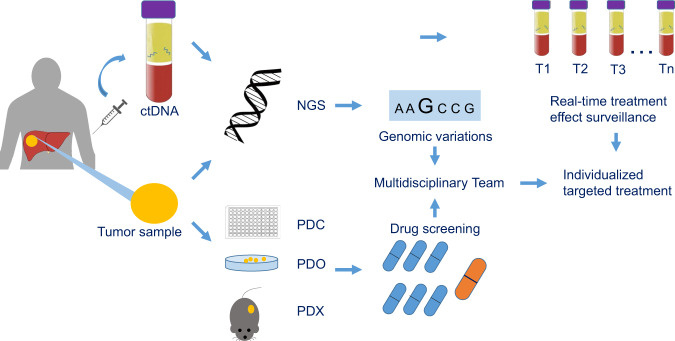
A schematic diagram of individualized targeted therapy integrated with novel technical platforms for HCC patients

Other approaches to understand the evolution of HCC and the mechanisms which underly the resistance to targeted therapy include next generation sequencing, the use of cancer organoids (PDOs) and PDX. PDO and PDX closely mimic hepatocarcinogenesis and preserve the tumor microenvironment making them excellent preclinical models for drug screening, biomarker development and research into the alterations and mechanism responsible for drug resistance.^225–227^ An example of this approach is our recent study using a patient-derived xenograft where we established 103 stable and transplantable xenograft lines that could be serially passaged, cryopreserved and revived. These lines maintained the diversity of HCC and the essential features of the original specimens at the histological, transcriptome, proteomic, and genomic levels. Using this model, we explored the predictive markers for sorafenib response and found that mitogen-activated protein kinase kinase kinase 1 (MAP3K1) might play an important role in sorafenib resistance and sorafenib response is impaired in patients with MAP3K1 down-expression.^228^ The combination of ctDNA sampling, next generation sequencing and PDX modeling has been applied to the clinical management of melanoma and facilitated personalized treatment.^229^ This approach using combinations of investigative techniques may also be applicable in the management of HCC and allow us to improve our understanding of the problems and opportunities for targeted therapy, tumor clonal evolution and interactions and facilitate the implementation of precision, personalized treatment (Fig. 3).

### Unsolved issues in targeted therapy for HCC

**Fig. 4:**
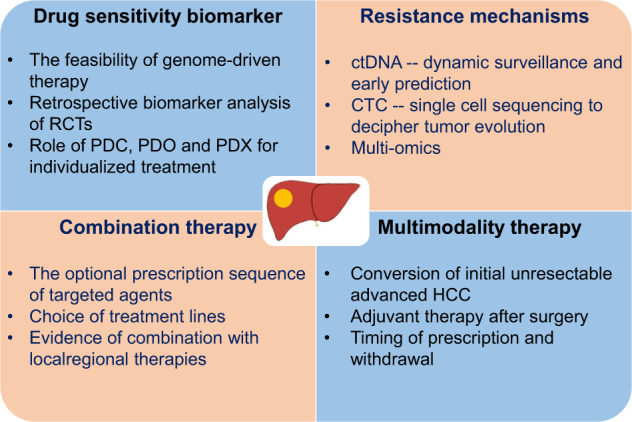
Future prospects of the targeted therapy for HCC

Several issues remain to be determined in the near future (Fig. 4). The best treatment strategy is still not clear, especially which targeted agent is the most appropriate for a specific HCC patient cohort. In the past, patients were faced with the harsh reality of knowing that other than sorafenib no therapeutic candidates were available. Today, a number of first and second-line options are available to clinicians. But in real-world medicine and most health care systems around the world, the majority of oncologist have to take into account the economic consequences before prescribing a particular treatment. Medical reimbursement or economic issues unfortunately remains an unavoidable consideration which influences decisions about treatment and the lack of our ability to provide clear guidance in respect of targeted treatment due to the myriad issues producing resistance to most therapies (*vide supra*) further complicates these decisions. We propose that the discovery and validation of novel biomarkers to reliably predict the response to treatment and define suitable candidates for a specific targeted agent, improving response rates and limiting avoidable toxicity in those who are unlikely to benefit, should be the focus of future HCC research.

In the clinical setting, we should ensure that we adhere to established treatment protocols particularly in respect of sequential targeted therapies or when a change of therapeutic agent is indicated. It is not uncommon for patients to be switched from sorafenib to lenvatinib, from regorafenib to lenvatinib, or from sorafenib to lenvatinib and to regorafenib when drug resistance develops, and these changes may be instigated by an oncologist or the patient themselves. While many patients do clearly benefit from these treatment changes, there is no evidence base underpinning many of these changes and this practice is undesirable as it confounds data collection at best and potentially exposes the responsible clinician to criticism especially where serious adverse events occur.

## Conclusion

An iterative approach to targeted therapy has provided a wealth of data and encouraging results particularly in those patients where resistance to sorafenib develops. Although the understanding and management of HCC has changed dramatically due to the extensive basic and clinical research which has occurred over the last decade, HCC sadly remains a devastating disease which has a ubiquitous, enormous impact on health care systems across the world. The advances over this period however mean that patients can be comforted by the knowledge that there is a huge international effort underway from oncologists, hepatologists, and basic scientists to fully understand the mechanisms which are providing more rapid progress and ensure that the prognosis continues to improve.
